# Identification by the DArTseq method of the genetic origin of the *Coffea canephora* cultivated in Vietnam and Mexico

**DOI:** 10.1186/s12870-016-0933-y

**Published:** 2016-11-04

**Authors:** Andrea Garavito, Christophe Montagnon, Romain Guyot, Benoît Bertrand

**Affiliations:** 1 Present Address: Centro de Bioinformática y biología computacional de Colombia – BIOS, Ecoparque los Yarumos, Manizales, Caldas Colombia; 2 RD2 Vision, 60 rue du Carignan, 34270 Valflaunes, France; 3 CIRAD, IRD, Interactions plants - micro-organisms - environment (IPME), Montpellier University, 911 Avenue Agropolis, BP 64501, 34394 Montpellier, France

**Keywords:** Genetic diversity, DArTseq, *Coffea canephora*, Mexico, Vietnam

## Abstract

**Background:**

The coffee species *Coffea canephora* is commercially identified as “Conilon” when produced in Brazil, or “Robusta” when produced elsewhere in the world. It represents approximately 40 % of coffee production worldwide. While the genetic diversity of wild *C. canephora* has been well studied in the past, only few studies have addressed the genetic diversity of currently cultivated varieties around the globe. Vietnam is the largest Robusta producer in the world, while Mexico is the only Latin American country, besides Brazil, that has a significant Robusta production. Knowledge of the genetic origin of Robusta cultivated varieties in countries as important as Vietnam and Mexico is therefore of high interest.

**Results:**

Through the use of Sequencing-based diversity array technology-DArTseq method-on a collection of *C. canephora* composed of known accessions and accessions cultivated in Vietnam and Mexico, 4,021 polymorphic SNPs were identified. We used a multivariate analysis using SNP data from reference accessions in order to confirm and further fine-tune the genetic diversity of *C. canephora*. Also, by interpolating the data obtained for the varieties from Vietnam and Mexico, we determined that they are closely related to each other, and identified that their genetic origin is the Robusta Congo – Uganda group.

**Conclusions:**

The genetic characterization based on SNP markers of the varieties grown throughout the world, increased our knowledge on the genetic diversity of *C. canephora*, and contributed to the understanding of the genetic background of varieties from very important coffee producers. Given the common genetic origin of the Robusta varieties cultivated in Vietnam, Mexico and Uganda, and the similar characteristics of climatic areas and relatively high altitude where they are grown, we can state that the Vietnamese and the Mexican Robusta have the same genetic potential to produce good cup quality.

**Electronic supplementary material:**

The online version of this article (doi:10.1186/s12870-016-0933-y) contains supplementary material, which is available to authorized users.

## Background

Canephora coffee produced by the coffee species *Coffea canephora* is named either “Conilon” when produced in Brazil, or “Robusta” when produced elsewhere in the world. In 2014, Canephora (hence Conilon and Robusta) coffee represented around 40 % of coffee production worldwide, while the remaining part corresponded to (http://www.ico.org/).

*C. canephora* is a rubiaceous plant originated from the sub-equatorial plains of Africa. It belongs to the *Coffea* genus, which comprises 124 species, originating from Africa, Madagascar, the Mascarene Islands, Asia and Oceania [1]. *C. canephora* and *Coffea* species are lowland, generally allogamous and diploids (2n = 2x = 22), with the notable exception of the highland, self-fertilizing allotetraploid (2n = 4x = 44) *C. arabica* [2]. Wild *C. canephora* plants are naturally distributed within intertropical Africa, stretching from Guinea to Uganda and from Central African Republic to Angola. Natural populations are composed of few individuals, subjected to gene flows from neighboring populations up to a few kilometers away [3, 4].

Based on former genetic studies [5–7], five main regions of wild genetically distant populations can be recognized: (i) West Africa (Guinea and Ivory Coast); (ii) Central Africa, Cameroon and Congo; (iii) the Atlantic frontage from Gabon to Angola; (iv) the Congo central basin; and (v) Uganda. The genetic diversity of *C. canephora* has been analyzed using isozyme markers [8, 9], microsatellites [10–12] and RFLPs [7, 13]. While these former analyses gave consistent results regarding the number and geographic origin of genetic groups, each independent work gave different names ending up with some confusion for the coffee community, suggesting the importance of precisely defining a general nomenclature. In this paper, we have therefore chosen, for clarity’s sake, the use of a new unified nomenclature for the five previously referenced genetic groups of *C. canephora,* which will be explained in detail in the plant materials section.

Whereas *C. arabica* was cultivated early (since the XIV^th^ century) in Ethiopia and Yemen, *C. canephora* cultivation dates back to the end of the XIX^th^ century, based on the use of local landraces populations. *C. canephora* was introduced to the main current producers of Robusta coffee by colonists during the 19^th^ century [14]. Until recently, it was thought that most of the cultivated *C. canephora* trees were derived from common sources reported to belong to the Congo basin [15, 16]. While former genetic diversity studies of *C. canephora* have focused on wild accessions from Africa and several Brazilian cultivated varieties [17], nothing is known about the genetic origin of coffee cultivated in Robusta-producing countries as important as Vietnam and Mexico. Vietnam is the first *C. canephora* producer (http://faostat3.fao.org), yet the genetic origin of the coffee plants grown by more than 400 000 cultivators in over 600 000 ha, within relatively high altitudes for Robusta coffee (>600 m.a.s.l), remains unknown. From 2012 to 2015, Vietnam produced 23 to 27 million 60 kg-bags of coffee, while Brazil produced 43 to 51 million of Arabica and Robusta taken together. In Latin America, apart from Brazil, only Mexico has a significant *C. canephora* production, producing 3.5 to 4.3 million (http://www.ico.org/). The qualities of the beans from Mexico and Vietnam have limited their marketability. Notably, Vietnamese beans are typically used in cheap soluble Western coffee. As a consequence of climate change, *C. arabica* growing will be affected in hotter lower (600–800 m.a.s.l.) production zones [18]. *C. canephora* could thus represent a good alternative for millions of small coffee farmers. In the near future, Mexican *C. canephora* varieties will probably become the sources of varieties for Central America, where *C. canephora* cultivation is rapidly expanding due to its resistance to several diseases. Knowing the genetic origin of the accessions cultivated in Vietnam and Mexico is therefore of the greatest interest.

As mentioned before, *C. canephora* genetic diversity has been analyzed using a limited number of isozyme, SSR and RFLP markers, representing only a restricted fraction of the *C. canephora* genome. In contrast to classical molecular markers, SNPs (Single nucleotide polymorphisms) are the most abundant markers, particularly in the non-coding regions of the genome [19]. New sequencing technologies (so called Next generation sequencing or NGS) used jointly with different complexity reduction methods, like the ones used in RADseq (Restriction site associated DNA sequencing) [20], GBS (Genotyping by sequencing) [21] and DArTseq (Sequencing-based diversity array technology) [22] methods, enable a large-scale discovery of SNPs in a wide variety of non-model organisms. When such techniques are applied to hundreds of genotypes, they provide measures of genetic divergence and genetic diversity within the major genetic clusters that comprise crop germplasm [23]. Indeed, the recently sequenced and assembled *C. canephora* genome, representing 64 % of the 710 Mb genome [24], facilitates the use of such marker technology and further analyses of the obtained data.

For this new extended study of the genetic diversity of *C. canephora*, we report the use of SNPs markers. In this study, DArTseq [22], a technique based on complexity reduction by the use of restriction enzymes targeting gene-rich regions and NGS sequencing, was used to study the genetic diversity of *C. canephora*. The specific objectives of the present study are (i) to test the performance of DArTseq method-derived markers in coffee: repeatability, error rates and genome wide representation of the markers; (ii) to assess consistency of *C. canephora* genetic diversity structures as compared to previous studies with ancient markers; and (iii) to identify the genetic origin of the coffee plants cultivated in Vietnam and Mexico, and to discuss possible consequences for coffee quality and breeding. By evaluating DArTseq-derived SNP markers from a set of well-known and unknown *C. canephora* accessions, it was possible to confirm and further fine-tune the genetic diversity of *C. canephora,* and to identify the genetic origin of accessions cultivated in two climate change susceptible zones, Vietnam and Mexico.

## Methods

### Plant material

Since each previous independent study has given different names to the genetic groups found, in this paper we have therefore chosen the use of the following nomenclature for the five previously referenced genetic groups of *C. canephora*: (i) “Guinean” Group (sometimes called D group), it is the genetic group originating from the Ivory Coast-Guinea area in West Africa; (ii) “Nana” group (sometimes called C group), stands for the coffee originating from the fringes of South-East Cameroon, South-West Central Africa and Northern Congo; (iii) “Conilon” group (sometimes called SG1 or A) represented by the Luki, Niaouli and Kouilou domesticated populations, originating from the south of Gabon; (iv) “Robusta Congo-Central Africa” group (sometimes called B), constituted by the wild coffees from the north of the Congo central basin and the south of Central Africa; and (v) “Robusta Congo-Uganda” group (sometimes called SG2) corresponding to the wild populations or cultivated varieties native to Uganda and the Congo basin.

A collection of 105 individuals from 87 accessions of *C. canephora* was analyzed in this study, from which 81 were used to analyze the diversity structure present in *C. canephora*. Known accessions, provided by the IRD (Institut de recherche pour le développement), were used as biological and technical replicates, to structure *C. canephora* diversity; while lyophilized leaves of plants cultivated in Mexico and Vietnam were supplied by AMSA (Agroindustrias unidas de México). Details on the accessions are given in Table 1 and Additional file 1: Table S1. *C. canephora* accessions are coded using the following rules: The first letter depicts their agronomical interest: wild (W) or cultivated (C). The following two letters represent their country of origin: Central African Republic (Ca), Congo (Cg), Ivory Coast (Ci), Cameroon (Cm), Uganda (Ug), Mexico (Mx), and Vietnam (Vn). The remaining numbers correspond to the plant number. Full siblings are named with “_” followed by the corresponding number. Biological replicates are named with “-” followed by the corresponding number. Accessions with technical replicates are marked as “-a” or “-b”.

**Table 1:** List of *C. canephora* accessions evaluated with DArTseq SNP markers

	Wild/cultivated	Origin (prospection or cultivated)	No. of individuals	Provider	Putative genetic group	Reference	Markers
Active individuals	Wild	South - East Cameroon	9	IRD	Nana	[7]	RFLP
	Wild	South - West Central African Republic	5	IRD	Nana	[7]	RFLP
	Wild	South Central African Republic	9	IRD	Robusta Congo-Central Africa	[7]	RFLP
	Wild/Cultivated	Ivory Coast	3	IRD	Guinean	[7]	RFLP
	Cultivated	Ivory Coast	2	CIRAD	Conilon	[35]	Isozymes
	Cultivated	Central America	2	Catie	Robusta Congo-Uganda	[10]	SSR
	Cultivated	Uganda	4	Cori, Uganda	Robusta Congo-Uganda	[10]	SSR
Subtotal			34				
Supplemental Individuals	Cultivated	Vietnam	6	AMSA	Unknown		
	Cultivated	Mexico, Chiapas	41	AMSA	Unknown		
Subtotal			47				
Biological replicates	Wild/Cultivated	Various	20	IRD/ CIRAD	Various		
Additional technical replicates	Wild/Cultivated	Various	4	IRD/CIRAD	Various		
Total			105				

Active individuals in multivariate analysis are those whose putative genetic group could be deduced from past studies. Other individuals, whose genetic group was unknown, were considered as supplementary individuals. Biological replicates are plants representing four different accessions

### DNA extraction and genotyping

Genomic DNA was extracted from leafs using the ADNid method (http://www.adnid.fr/index-2-4A.html). Technical replicates from two independent DNA extractions were used for some accessions and several accessions were represented by more than one tree, as biological replicates (Additional file 1: Table S1). Genotyping was carried out at DArT P/L in Canberra-Australia, using a combination of HiSeq 2000 (Illumina) next-generation sequencing with DArT technology, as previously described [22]. The SNP markers obtained were used for data analysis after discarding markers with more than 10 % of missing data and a minor allele frequency (MAF) below 1 %.

### Data analysis

In order to obtain the genotyping error rates of the DArTseq method when applied to coffee, the identical allele call rates in technical and biological replicates were evaluated with the “Similarity of Individuals” function from the Joinmap 4.1 software [25], based on SNPs with no missing data within the entire panel of replicates. Then, the error rates were calculated as the number of allelic differences between replicates, divided by the total number of markers analyzed [26].

All the genetic statistical analyses were carried using R, version 3.2.3 [27]. The polymorphic information content (PIC) for each SNP marker was calculated using the equation $PIC=1-{\sum}_{i=1}^{\mathrm{n}}{p}_i^2$ with p^2^_i_ representing the squared frequency of allele *i* at each locus. Statistics such as the mean observed heterozygosity (Ho), and mean expected heterozygosity (He) were calculated with the “adegenet*”* 2.0.2 package [28]. The Fixation index (F_ST_) was calculated with the “fstat” function of the “hierfstat” 0.04–22 pakage [29]. The percentage of missing data and MAF were calculated using the “SRPRelate” 1.4.2 package [30]. Diversity structure present in the *C. canephora* collection was analyzed using a Discriminant Analysis of Principal Components (DAPC) multivariate analysis implemented in “adegenet” [31], as follows: First, 34 known individuals (Table 1) corresponding to the previously described diversity groups [10, 32] were used to model the diversity present in the panel, after centering the data. The most probable number of groups that define the diversity evaluated were inferred using the “find.cluster” function, running successive K-means with an increasing number of clusters (k) from one to ten, and with the Bayesian Information Criterion (BIC) as the statistical measure of goodness of fit. The number of retained Principal Components (PC) to be used in the discriminant analysis was determined using the “xvalDapc” function with the default parameters. Second, individuals with a probability of membership over 80 % to each genetic group were subjected to another round of DAPC analysis in order to find possible subgroups, following the same procedure. Using a threshold calculated with the median hierarchical clustering method implemented in the “snpzip” function from “adegenet”, a set of alleles with the highest contribution to the between-population structure was identified. Additionally, we used the outlier test based on the joint distributions of expected heterozygosity and F_ST_ under an island model of migration, implemented in LOSITAN [33], in order to identify the SNP loci under selection and to compare them to the ones discriminating the genetic groups identified. A first run consisting of 100,000 simulations was used to remove outlier candidate SNPs outside the 99 % confidence interval. A neutral F_ST_ value was then recalculated, and with it, outlier SNPs were identified after 100,000 simulations, as the ones outside the 1 to 99 % confidence interval, with a false discovery rate smaller than 0.05.

Finally, individuals of unknown groups were projected onto the discriminant functions found with DAPC, using the “predict” function from the package.

To illustrate the genetic relationships between individuals, unrooted NJ trees were constructed with the package “poppr” 2.1.0 [34], based on a Nei’s genetic distance matrix, modified to measure distances between individuals. Bootstrap analyses were also computed with “poppr”*,* using 100 iterations.

### Sequence comparisons

The sequences obtained by the DArTseq method, containing the filtered SNPs markers, were mapped against *C. canephora* pseudo-molecules [24] and predicted *C. canephora* genes (available at http://coffee-genome.org), using the Bowtie2 algorithm [35] with the very sensitive, end-to-end alignment option. Markers with the highest contribution to the between-population structure were similarly mapped on the *C. canephora* pseudo-molecules and genes. Graphical representations of the hits were drawn with the “Circos” program [36].

## Results

### Marker descriptions and distribution

After sequencing 105 individuals from *C. canephora*, we obtained 10,806 DArTseq-derived SNP markers*.* The average missing data and MAF percentages were 16.3 % and 12.8 %, respectively. After removing markers with more than 10 % of missing data and MAF below 1 %, 4,021 polymorphic SNPs remained for the analysis, with an average missing data of 3.1 %, a MAF percentage of 12.6 %, and an average PIC of 0.159 for the whole sample panel. The mean Ho and mean He calculated for the 4,021 markers were 0.124 and 0.162, respectively, estimated based on a panel of depurated biological and technical replicates (81 unique accessions) in order to avoid any bias on the measure.

**Fig. 1:**
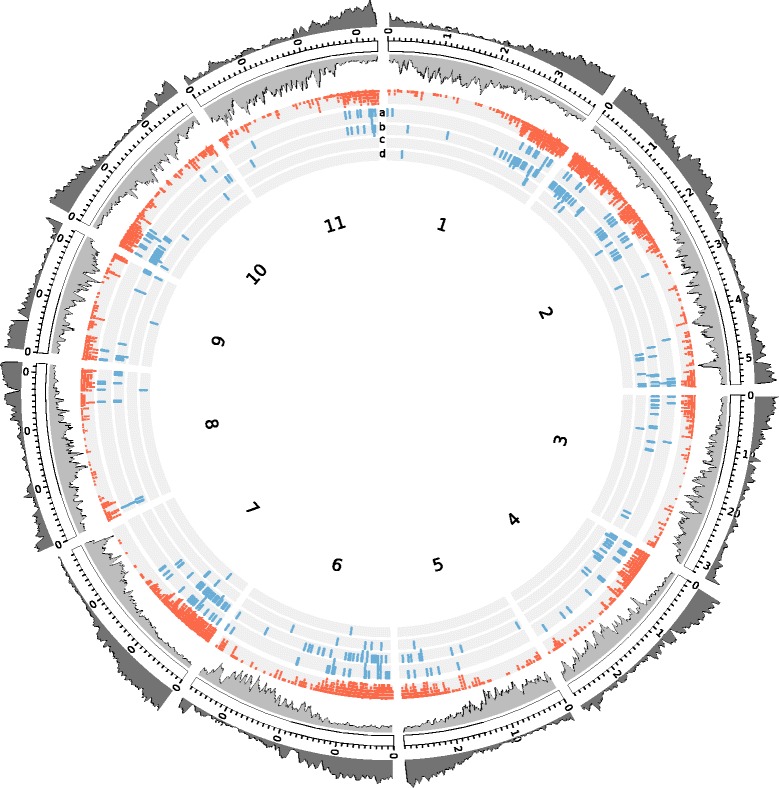
Distribution of DArTseq-derived SNP markers in the *C. canephora* genome. Graphical representation of the eleven pseudo-molecules of *C. canephora* showing the density of genes (*dark gray*) and transposable elements (*light gray*), along with the location of the 4,021 DArTseq SNP markers used for the analysis (*red*). Markers with the highest contribution (*blue*) to the first (**a**), second (**b**), third (**c**) and fourth (**d**) discriminant axes deciphering the genetic structure of *C. canephora* are also shown

The 4,021 DArTseq-derived SNP markers were obtained from 3,388 unique sequences (Additional file 1: Table S2). These sequences showed a tendency towards gene-rich regions when mapped on the recently sequenced *C. canephora* genome (Fig. 1), with 90.8 % of sequences aligned on the pseudo-molecules, and 35.7 % within annotated gene sequences. The average density in the genome was one marker per 178 kb.

Technical and biological replicates allowed us to assess the reliability of the DArTseq method in coffee. Genotyping error rates in technical and biological replicates for the 2,616 SNPs with no missing data within the entire panel of replicates were 4.0 % (s = 1.0) and 4.3 % (s = 0.8), respectively Additional file 2: Figure S1. The difference between the two types of replicates was not significant (*p*-value = 0.2887). Taken together, these results suggest that the overall error rate in allele calls for the DArTseq method in *C. canephora* would be near 4 %.

### Genetic structure of the *C. canephora* collection

The observed and expected heterozygosities calculated with 4,021 SNPs for the 34 analyzed accessions were 0.1405 and 0.1933, respectively (Table 2).

**Table 2:** Observed and expected heterozygosities found for the five *C. canephora* genetic groups

	Group1	Group 2	Group 3	Group 4	Group 5	Total
Ho	0.1360	0.1641	0.1215	0.0530	0.1347	0.1405
He	0.1199	0.1642	0.1007	0.0456	0.1283	0.1933

In order to interpret *C. canephora* diversity in a whole genome context, the DArTseq SNP data obtained from a collection of 34 *C. canephora* members of previously known diversity groups was analyzed using a DAPC multivariate analysis.

**Fig. 2:**
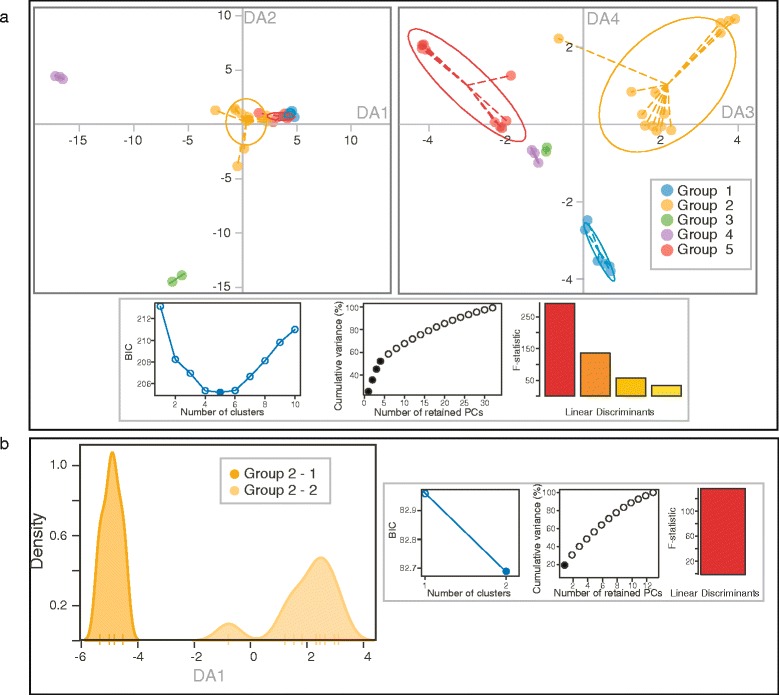
Genetic structure of *C. canephora* individuals evaluated with 4,021 DArTseq SNP markers. Scatter plots from the DAPC analysis carried out with 34 *C. canephora* accessions. **a** Discriminant axes 1 and 2 (left) and 3 and 4 (right) representing the five groups (inertia ellipses) determined by the DAPC. Group 1 encloses cultivated individuals from Congo and Uganda, known to belong to the Robusta Congo-Uganda group; Group 2 represents the accessions previously described into the Nana group, from Cameroon and the Central African Republic; Group 3 is equivalent to the Conilon group, with cultivated individuals from the Ivory Coast; Group 4 is made up of only wild and cultivated Guinean accessions collected in the Ivory Coast; and finally, Group 5 is composed of wild individuals from the Central African Republic belonging to the Robusta Congo-Central Africa group. **b** First discriminant axis deciphering the genetic relationships between individuals from the two sub-groups of group 2. For each DAPC analysis (**a** and **b**), the Bayesian information criterion (BIC) used to determine the optimal k number of clusters (blue dot), the percentage of cumulative variance for the retained PCA eigenvectors (black dots), and the F-statistic of the between/within group variance ratio for the discriminant functions (colored bars) are also exposed below each DAPC plot

The first four principal components of the principal component analysis (PCA), which explained 25.4 %, 10.3 %, 9.5 % and 7.0 % of the variance, respectively, were retained for the discriminant analysis with the DAPC function. Genetic diversity, as revealed by the DArTseq-derived SNP markers, confirms the genetic diversity previously revealed by RFLPs and SSRs, as five genetic clusters were identified (Fig. 2a). A detailed observation on the accessions belonging to the obtained groups allowed us to find equivalences, as follows: (i) Group 1 encloses cultivated individuals from Congo and Uganda, known to belong to the Robusta Congo-Uganda group; (ii) Group 2 represents the accessions previously described in the Nana group, from Cameroon and the Central African Republic; (iii) Group 3 is equivalent to the Conilon group, with cultivated individuals from the Ivory Coast; (iv) Group 4 is made up of only wild and cultivated Guinean accessions collected in the Ivory Coast; and finally, (v) Group 5 is composed of wild individuals from the Central African Republic belonging to the Robusta Congo-Central Africa group. The first discriminant axis of the DAPC clearly separates the Guinean and Conilon groups from the three others, while the second axis opposes the Conilon group against the rest of the groups. The third axis discriminates the Robusta Congo-Central Africa group from the Nana group; and the fourth axe separates the Robusta Congo-Uganda group from the others. The observed and expected heterozygosities estimated for the groups ranged from 0.0530 to 0.1641, and from 0.0456 to 0.1642, respectively (Table 2).

In order to identify the genomic regions contributing to the population structure found in *C. canephora,* the identity and genome location of the SNPs discriminating the five groups were determined, taking advantage of the recently available *C. canephora* genome [24]. Out of 149, 240, 33 and 8 structural alleles contributing to the four discriminating axes (Additional file 1: Table S3), respectively, 125, 205, 26, and 5 were mapped only once to the *C. canephora* genome; while 15, 17, 5 and 2 mapped more than once, and 54, 99, 12, and 2 fell into an annotated gene. Their putative functions and gene ontologies show a large range of putative functions (Additional file 1: Table S3), with a high representation of genes involved in signal transduction, and a higher distribution on gene-rich regions on the *C. canephora* pseudo-molecules (Fig. 1).

In order to identify SNP loci under selection and to compare them to the ones discriminating the genetic groups identified, an outlier test based on the joint distributions of expected heterozygosity and F_ST_ was used. An initial F_ST_ of 0.3307 was calculated based on the 4,021 markers. After candidates for outliers were removed, a simulated F_ST_ of 0.4815 was found. From the 4,021 SNPs, 793 were found to be under balancing selection, 107 under positive selection, while the rest was found to be under neutral selection (Additional file 1: Table S4, and Additional file 3: Figure S2). When comparing the discriminant markers identified by the DAPC analysis to the ones found by the outlier test, we found that 12.9 % (55 SNPs) are subject to positive selection, while the rest are under neutral selection (Additional file 1: Table S3).

In order to establish a more detailed structure of the species, a second DAPC analysis was carried out with groups containing a sufficient number of individuals. In this manner, a more profound genetic structure was found only for Group 2, with two subgroups (Fig. 2b). Group 2–1 includes all but one individuals from the south-western Central African Republic from the Nana group, and Group 2–2 consists of all the South-Eastern Cameroon individuals evaluated in the study.

Taken together, the present analysis corroborates the previous structure of the *C. canephora* diversity, and adds a higher level of resolution to the observed structure.

### Genetic structure of cultivated overseas accessions

**Fig. 3:**
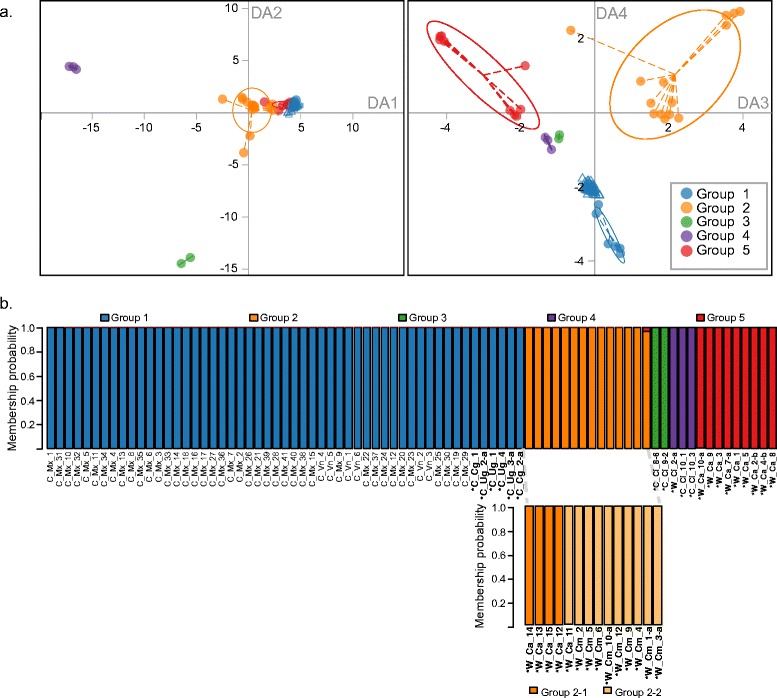
Genetic origin of *C. canephora* cultivated overseas accessions. Scatter plots from the DAPC analysis, showing the 81 *C. canephora* accessions analyzed. **a** Discriminant axes 1 and 2 (left) and 3 and 4 (right) representing the five groups (inertia ellipses) determined by the DAPC, as explained in Fig. 2. Empty circles represent reference accessions used to identify genetic groups, as in Fig. 2, while empty triangles represent interpolated individuals from Vietnam and Mexico. **b** Bar plots of the posterior membership probabilities obtained with the DAPC analysis. The top barplot represents the five groups found, while the bottom shows the sub-groups derived from group 2. The names of the 34 accessions used to identify the genetic groups are highlighted with an (*), and written in bold characters

With the aim of assessing group membership of cultivated accessions in Vietnam and Mexico and to identify their putative origin, the DArTseq SNP data obtained from the evaluation of 47 additional *C. canephora* accessions were interpolated into the DAPC analysis (Fig. 3a). All newly incorporated accessions collocated closely with individuals of the Robusta Congo-Uganda group. Membership probabilities for each accession were close to 100 % (Fig. 3b).

**Fig. 4:**
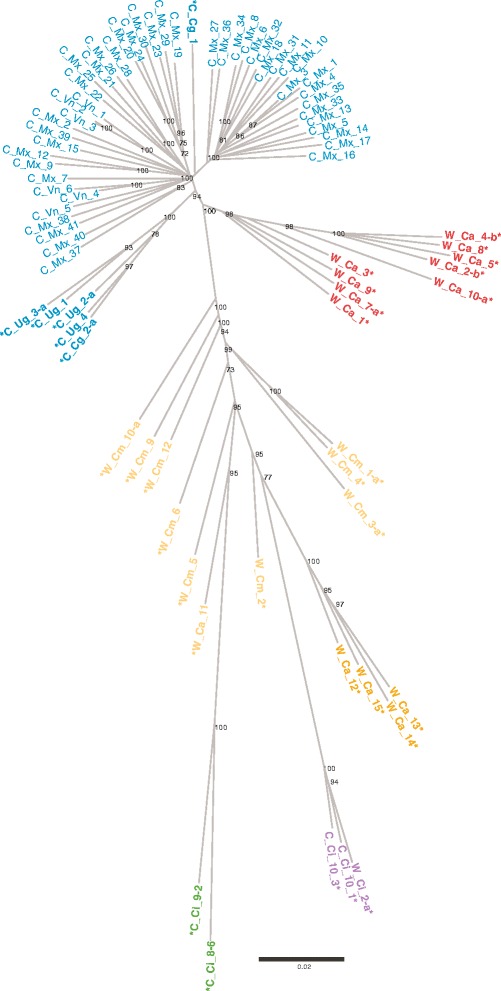
Neighbor Joining tree based on SNP marker evaluations. Unrooted tree using the Neighbor-joining algorithm based on Nei’s genetic distances between 81 individuals of *C. canephora*. Accessions marked with an (*) are active individuals used in the DAPC analysis to determine the genetic groups. The color patterns are equivalent to the barplots in Figs. 2 and 3, where blue represents cultivated individuals from Congo, Uganda, Vietnam and Mexico, known to belong to the Robusta Congo – Uganda group; Orange and yellow represent the accessions previously described into the Nana group, from Cameroon and the Central African Republic; Green is equivalent to the Conilon group; purple represents wild and cultivated Guinean accessions collected in the Ivory Coast; and finally, red represents wild individuals from the Central African Republic belonging to the Robusta Congo-Central Africa group. For clarity’s sake only bootstrap values over 70 are exposed

In order to obtain a more complete picture of the genetic relationships linking the *C. canephora* accessions evaluated in the present study, a NJ tree was constructed using the 4,021 SNP markers (Fig. 4). The tree comprises at least eight well-defined branches, all in agreement with the DAPC results. Two branches encompass the Vietnamese and Mexican accessions from the Robusta Congo-Uganda group, as well as one Congolese accession; another branch includes the Ugandan and one Congolese individuals from the same genetic group; at least one branch encompasses the Robusta Congo-Central Africa group; at least two correspond to the Nana group; and there is one branch for each of the Guinean and the Conilon groups.

## Discussion

In the present study, we have employed a DArTseq method on a *C. canephora* collection. After evaluation, we found an overall genotyping error for the obtained SNP markers close to 4 %, which is similar to what has been previously reported for NGS derived data [37]. The number of exploitable SNPs, repeatability and missing data is similar to what has been obtained using the same technique with other crops [22, 38, 39]. The obtained SNP markers seem to be located mostly in gene-rich parts of the genome, making them an excellent resource for traditional gene mapping or even association mapping assays in coffee trees. The DArTseq method is therefore particularly reliable and easy to use as part of genetic diversity studies. Also, the implementation of these markers in germplasm collections represents an appreciable tool for the curation and optimization of such resources, as it enables a simple means for eliminating redundant or mistagged accessions. From our analysis, we found Ho and He not very distant from the ones calculated previously with microsatellites [5] when evaluated for the complete reference panel, while the observed and expected heterozygosity estimates for the groups were almost half of what has been observed in the past in *C. canephora* groups using microsatellites [5]. In addition, the data obtained in the present study has allowed us to decipher the diversity of *C. canephora* in a genome-wide context, and to identify the possible origin of several cultivated accessions from countries where *C. canephora* has a crucial economic importance. Our *C. canephora* genetic diversity analysis soundly supports previous studies based on a restricted number of molecular markers [7–13], with all groups unambiguously identified using the DArTseq-derived SNP markers. Compared to former analyses, our study provides a better characterization of the Nana group, through subgroups: one composed of accessions from Southeastern Cameroon and the other from Southwestern Central African Republic. It is clear that a more complete collection evaluated with SNPs derived from one of the NGS technologies would give a better look of the species diversity, especially for groups that were under-represented in our analysis.

By comparing the 427 unique discriminant SNPs identified by the DAPC analysis with the outliers found based on the joint distributions of expected heterozygosity and F_ST,_ we were able to infer that nearly 87 % of the differential alleles found with the DAPC analysis seem to have been fixed randomly within the populations. The remaining discriminant alleles found to be under positive selection may have been differentially fixed in the populations as an adaptation to local environmental conditions encountered at the sites of origin of each group.

Although it is not possible to ensure whether all the identified differential alleles are actively or directly involved in the evolutionary differentiation between the groups, or whether they are simply highly linked to the actual causal factor, it is still interesting to seek out the putative molecular function of the genes in which they reside. Most of the markers are located in annotated genes coding for proteins involved in signal transduction, while others reside in proteins constituting cellular organelles, and even DNA-interacting proteins.

In contrast with the *C. canephora* cultivated trees from Brazil, which originated mainly from the Conilon group [17], here we revealed for the first time that Mexican and Vietnamese *C. canephora* cultivars form a cluster with the “Robusta Congo-Uganda group”. The genetic origin of populations grown in Mexico and Vietnam appears to be the same as that of Ugandan cultivars, for which Cubry and coworkers [10] showed that they were not distinguishable from wild Ugandan *C. canephora* individuals. Therefore, the genetic basis introduced in Vietnam, Mexico, and Brazil reflects the wild African genetic groups from where they are originated, indicating that the two main producers of Robusta coffee in the world (i.e., Vietnam and Brazil) produce beans from two very different genetic origins.

In Vietnam as well as in Mexico and Uganda, cultivated *C. canephora* trees are grown at relatively high altitudes (>600 m.a.s.l.), as compared to the usual 0–400 m.a.s.l range [40] used elsewhere. It is interesting to note that in Mexico and Vietnam coffee trees are distributed over the same latitude range (Latitude: 12.00° N to 20.00° N). In both countries, the optimum coffee-producing zone is at an altitude between 300 and 900 m.a.s.l. In Uganda, the same coffee group is grown near the equator between 300 and 1,100 m. This data suggests that there is a wide adaptability of the “Robusta Congo-Uganda group”, since it is able to adapt in mountainous areas with rather cool climates and fairly high latitude areas, as well as in low-lying areas and low latitudes. This is also observed in Indonesia (the third biggest Robusta producer) that grows coffee from the same genetic group at latitudes ranging between 5 and 11° latitude to 300 to 1,200 m.a.s.l. Since Robusta coffee produced in Uganda has a very good reputation in terms of quality, we can deduce that the relatively bad reputation of Robusta produced in Vietnam (in intensive and full-sun systems), and in a lesser extent in Mexico and Indonesia (in extensive and agroforestry systems), is probably mainly due to poor quality of post-harvest treatments.

In the long term, climate changes-particularly, global warming-will affect not only the three biggest producing countries (i.e., Vietnam, Indonesia and Brazil), but also several producing countries like Mexico. Is the “Conilon” genetic group present in Brazil more adapted to climate change than the “Robusta Congo-Uganda group” present in Asia or Mexico? This issue needs to be addressed by researchers to predict supply scenarios for the industry and growers. We strongly recommend comparing the performance of Robusta to Conilon cultivars under abiotic stresses. We also suggest comparing those origins with hybrids produced between genetics groups.

In the majority of Robusta-producing countries, the current genetic diversity available for breeding programs is very low [41]. The introduction of a core collection representing the genetic diversity of the species is a priority for breeding programs in a climate change context. Thus, a similar initiative to that implemented by the World Coffee Research (http://www.ico.org/) for Arabica should be undertaken urgently for *C. canephora*, in order to cope with future challenges brought about by the evolving climate conditions.

## Conclusions

In the present study, we established that markers obtained from NGS approaches are easily exploitable in coffee, with an error rate similar to what has been observed for other crops. The genetic characterization based on SNP markers of the varieties grown throughout the world increased our knowledge on the genetic diversity of *C. canephora*, and contributed to the understanding of the genetic background of varieties from very important coffee producers. Also, the discriminant SNP markers identified in our work represent a valuable tool that could be used by breeders to discriminate between *C. canephora* genetic groups in Robusta germplasm.

The quality of Mexico and Vietnamese coffee are traded at a price lower than Uganda. Given the similar characteristics of climatic areas and relatively high altitude where Robusta is grown in the three countries, and given the common genetic origin of the varieties cultivated, we can state that the Vietnamese and Mexican Robusta accessions have the genetic potential to increase the quality of Robusta they produce.

## Additional files

Additional file 1: Table S1. List of plants evaluated with DarTseq markers. **Table S2.** List of DArTseq markers and used in the analysis. **Table S3.** Structural SNPs contributing to the population structure in *C. canephora*. **Table S4.** Putative outlier marker loci identified by the Fst outlier method implemented in Lositan. (XLSX 457 kb)
Additional file 2: Figure S1. Genotyping error rates in technical and biological replicates. Genotyping error rates for 15 and 4 accessions with technical (**a**) and biological (**b**) replicates evaluated with SNPs. Ratios were obtained using all 4,021 markers (blue) or by selectively ignoring markers having differential missing data (red). (EPS 683 kb)
Additional file 3: Figure S2. Selection test for each of the 4021 DArTseq SNP markers in *C. canephora* Plotted distribution of the empirical F_ST_ values versus the expected heterozygosity. The red and blue lines indicate 99 % and 1 % confidence limits, respectively, while the gray line corresponds to the median value. (PDF 192 kb)

## Abbreviations

AMSA: Agroindustrias Unidas de México
BIC: Bayesian Information Criterion
CIRAD: Centre de coopération internationale en recherche agronomique pour le développement / French agricultural research and international cooperation organization
DA: Discriminant axis
DAPC: Discriminant analysis of principal components
DArTseq: Sequencing-based diversity array technology
GBS: Genotyping by sequencing
He: Mean expected heterozygosity
HL: Homozygocity by loci
Ho: Mean observed heterozygosity
INEAC: Institut national d’études agronomiques du Congo Belge
IRD: Institut de recherche pour le développement
m.a.s.l: Meters above sea level
MAF: Minor allele frequencies
Na: Mean allele number
NGS: Next generation sequencing
NJ: Neighbor-joining
PC: Principal component
PCA: Principal component analysis
PCR: Polymerase chain reaction
PIC: Polymorphic information content
RADseq: Restriction site associated DNA sequencing
RFLPs: Restriction fragment length polymorphism
SNPs: Single nucleotide polymorphism
WCR: World coffee research
