# Supplementary figures and images for: Identification by the DArTseq method of the genetic origin of the Coffea canephora cultivated in Vietnam and Mexico
Source: BMC Plant Biol. 2016 Nov 4;16:242. doi: 10.1186/s12870-016-0933-y (PMC5096298; doi:10.1186/s12870-016-0933-y)

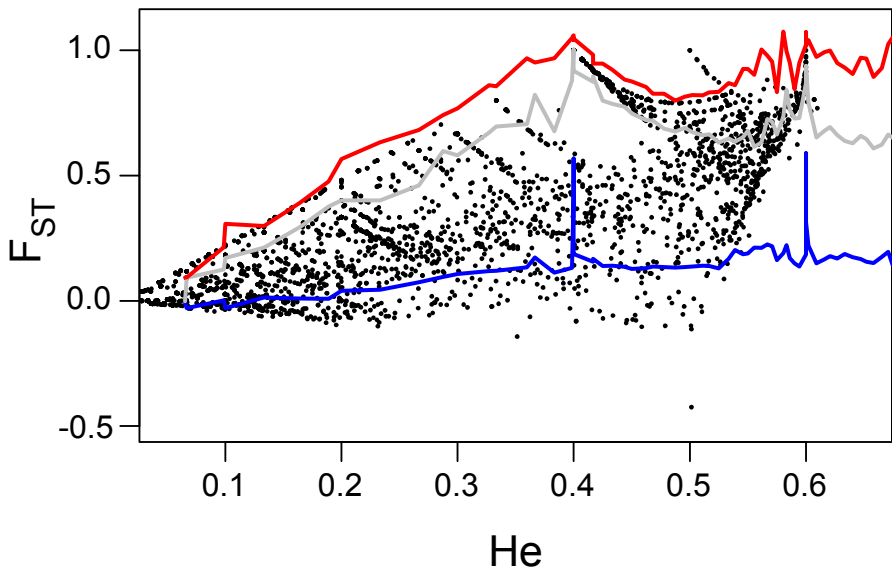

Supplement: Additional file 3: Figure S2. — Selection test for each of the 4021 DArTseq SNP markers in C. canephora Plotted distribution of the empirical FST values versus the expected heterozygosity. The red and blue lines indicate 99 % and 1 % confidence limits, respectively, while the gray line corresponds to the median value. (PDF 192 kb) [file 12870_2016_933_MOESM3_ESM.pdf]
